# Ballistic head and neck injuries: management rationale and outcomes in a case series

**DOI:** 10.1097/RC9.0000000000000741

**Published:** 2026-07-22

**Authors:** Ahmad Alkheder, Adel Azar, Ahmad Mustafa

**Affiliations:** a Department of Otorhinolaryngology, Al Mouwasat University Hospital, Damascus University, Damascus, Syria; b Faculty of Medicine, Damascus University, Damascus, Syria; c Faculty of Medicine, Syrian Private University, Damascus, Syria

**Keywords:** case series, head and neck, management, observation, penetrating injuries, surgery

## Abstract

**Introduction:**

Penetrating head and neck injuries with retained foreign bodies pose significant management challenges due to the anatomical density of vital structures. The optimal therapeutic strategy – ranging from surgical extraction to conservative observation – remains guided by individual patient and injury characteristics.

**Methods:**

This case series retrospectively reviews four distinct presentations of retained metallic foreign bodies in the head and neck region. Diagnostic evaluation utilized advanced computed tomography (CT) for precise localization. Management strategies were individualized, including functional endoscopic sinus surgery, open surgical exploration via parotid and cervical approaches, and active observation.

**Results:**

CT imaging was critical for preoperative planning in all symptomatic cases, accurately defining the relationship of fragments to neurovascular structures. Endoscopic retrieval was successful for a fragment within an Onodi cell. Open approaches were required for fragments embedded in the parotid region and deep neck, with one patient developing Frey syndrome postoperatively. In an asymptomatic patient with no structural compromise, observation was safely elected. Delayed complications, including chronic pain and infection, manifested years after the initial injury in two cases.

**Conclusions:**

Management of penetrating head and neck foreign bodies requires a tailored, multidisciplinary approach. Advanced imaging is indispensable for risk assessment and planning. Treatment should be individualized, with minimally invasive techniques preferred when feasible, while observation is a valid option in select asymptomatic patients. Long-term follow-up is essential due to the potential for delayed sequelae.

## Introduction

Penetrating injuries to the head and neck region represent a significant clinical challenge, owing to the anatomical complexity and high density of vital neurovascular structures in this area^[^1^]^. The management of these injuries is further complicated when foreign bodies are retained, as they can lead to a spectrum of delayed complications, including infection, chronic pain, neurological deficits, and inflammatory reactions^[^2,3^]^. The diagnostic process hinges on a high index of suspicion and relies heavily on advanced imaging techniques to precisely localize foreign objects and assess their relationship to critical anatomical landmarks^[^1,4^]^. The therapeutic approach has evolved from mandatory exploratory surgery to a more nuanced, case-specific strategy. This is largely due to advancements in radiological guidance and minimally invasive endoscopic techniques, which allow for a more conservative approach in selected stable patients, thereby minimizing surgical morbidity^[^5,6^]^. However, the decision between surgical intervention and conservative management must be carefully weighed, considering factors such as the material, size, and location of the foreign body, as well as the presence of symptoms or impending complications^[^1,3^]^.

HIGHLIGHTS

- Computed tomography is essential for accurately locating retained foreign bodies and facilitating effective preoperative planning, particularly in symptomatic patients.
- Functional endoscopic sinus surgery is the preferred minimally invasive technique for removing centrally located sinonasal foreign bodies, reducing morbidity, and preserving normal anatomy.
- For foreign bodies situated in deep or complex regions, open surgical approaches, such as Blair incisions, are employed, with meticulous preservation of nerves and blood vessels.
- Observation is a viable option in select cases; in asymptomatic patients with no structural damage, conservative observation was safely adopted, indicating that not all retained foreign bodies require surgical removal.
- The choice of removal technique depends on the foreign body’s location, symptomatology, and potential risks, emphasizing individualized management strategies.

This case series illustrates the diverse and complex nature of penetrating head and neck trauma with retained foreign bodies. We present four distinct cases that highlight the variability in presentation, the critical role of tailored imaging, and the application of different management strategies – from endoscopic retrieval and surgical exploration to active observation. Through these cases, we aim to underscore the importance of a multidisciplinary approach and meticulous preoperative planning in optimizing functional and aesthetic outcomes while mitigating the risks of long-term sequelae. This case series has been reported in line with the PROCESS 2025 guideline^[^7^]^.

## Methods

This retrospective case series reviews four patients with retained metallic foreign bodies in the head and neck region. Diagnostic assessment relied on computed tomography for precise anatomical localization. Management was individualized, spanning functional endoscopic sinus surgery, open surgical approaches, and active observation based on clinical and radiographic findings.

## Case presentation

### Case 1

A 39-year-old male sustained multiple injuries from explosive fragment trauma, including a retained foreign body. He presented with recurrent headaches and occasional seizures, along with signs of traumatic brain injury. There was no rhinorrhea. He had mild hearing impairment and multiple ballistic injuries. He also had a history of multiple abdominal surgeries and psychological disturbances following trauma. Otologic and oral examinations were normal. The nose showed a left deviation of the nasal septum with chronic mucosal inflammation. Computed tomography (CT) imaging revealed a foreign body between the anterior wall of the sphenoid sinus and the posterior ethmoid sinus, located within the right Onodi cell (Fig. 1).

**Figure 1.**
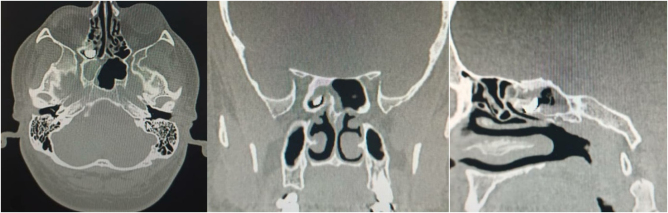
CT demonstrates a metallic foreign body between the anterior wall of the sphenoid bone and the posterior ethmoid sinus, within the right Onodi cell.

Functional endoscopic sinus surgery (FESS) was performed. Inflammatory polypoid formations were identified in the middle meatus, as well as the anterior and posterior ethmoids. These findings were not visible on the preoperative CT due to the two-year interval between the injury and surgery. The procedure included meatotomy, anterior and posterior ethmoidectomy, progressing to the location of the foreign body in the right Onodi cell. The foreign body was found between the anterior wall of the sphenoid sinus and the posterior ethmoid sinus, adjacent to the optic nerve canal, and residing within the right Onodi cell. It was successfully removed after multiple soft maneuvers without any injury to the right optic canal, which lay just lateral to the fragment. Finally, the right sphenoid orifice was enlarged to drain purulent secretions from the sinus. Mucosal inflammation in the sphenoid sinus was noted, which appeared after the extraction of the foreign body and sphenoidotomy (Fig. 2).

**Figure 2.**
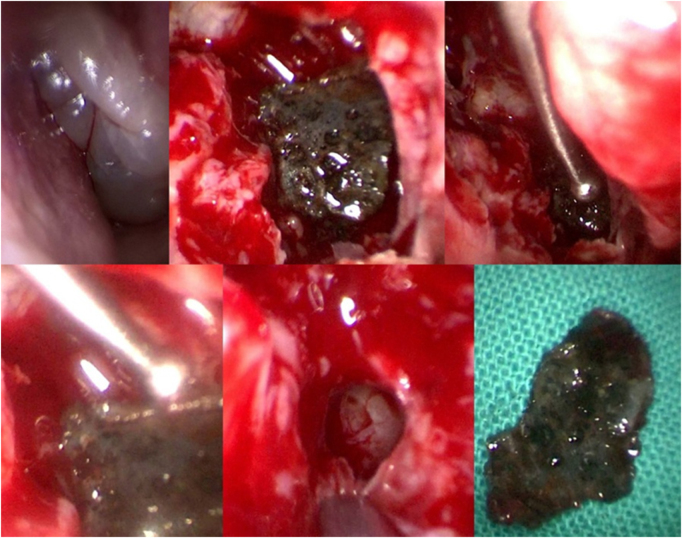
Extraction of the fragment during functional endoscopic sinus surgery.

### Case 2

A 32-year-old woman sustained a penetrating injury from a projectile fragment in the left parotid region. She was admitted to the maxillofacial surgery department at the time of the injury. Initial findings included a radiopaque foreign body located anterior and inferior to the coronoid process, with a comminuted defect in the surrounding tissues and a concomitant dental injury (Fig. 3). Neurologic examination showed involvement of the V3 (mandibular) distribution, with numbness extending from the left mandibular region to the mental region. Partial injury to the facial nerve resulted in mild weakness of some left-sided facial expressions. Intermaxillary fixation (maxillomandibular fixation) with arch-bar stabilization was planned for approximately 2 months.

**Figure 3.**
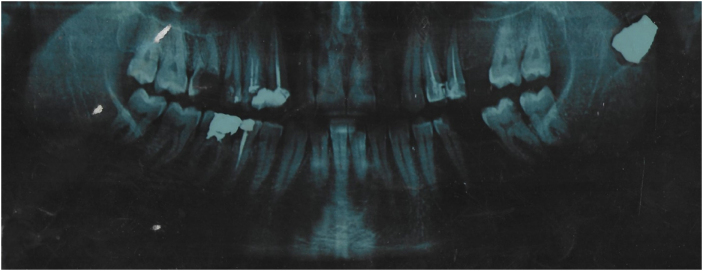
Three years post-stabilization of the mandible and maxilla: bone healing evident; radiopaque foreign body in the parotid region.

Seven years after the initial trauma, the patient began experiencing headaches and severe pain reminiscent of trigeminal neuralgia, requiring analgesic treatment. About 8 years post-injury, MSCT revealed a metallic foreign body measuring 15 × 19 × 20 mm, located inferior to the anterior portion of the zygomatic arch, anterior and inferior to the coronoid process of the mandible, at the medial-anterior superior margin of the masseter muscle (Fig. 4). The foreign body remained in place for years, with radiologic confirmation of its position. It was exposed and removed via a Blair incision (parotid approach) with extracapsular dissection. The Stensen’s duct, as well as the buccal and mandibular branches of the facial nerve, was identified and preserved; the foreign body was located between these branches (Fig. 5). The operation proceeded without intraoperative complications.

**Figure 4.**
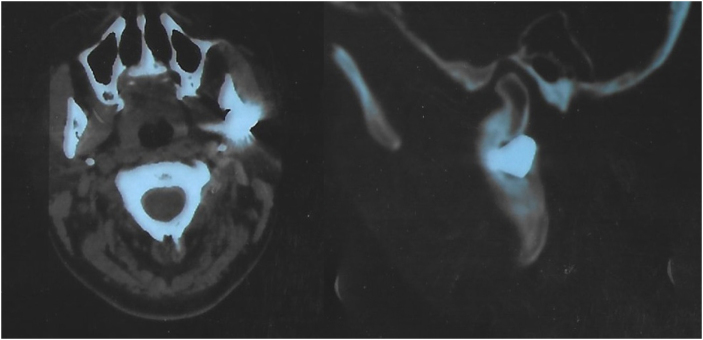
A computed tomography scan showing the location of the metallic foreign body.

**Figure 5.**
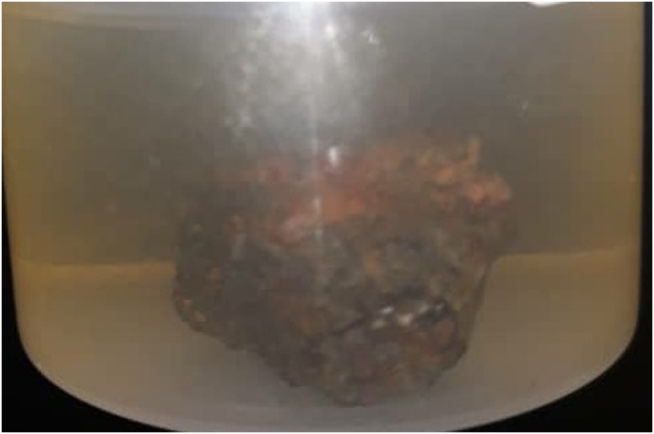
A military fragment after extraction.

The patient reported mild gustatory sweating about 1 year after removal, occurring in the anterior and inferior region of the auricular area during meals, and more pronounced when consuming acidic foods such as lemon. A non-contrast CT performed about 2 years later showed normal findings in the region (Fig. 6). A panoramic radiograph obtained around 3 years postoperatively demonstrated the postoperative appearance (Fig. 7). A follow-up photograph taken about 4 years after surgery documented the long-term appearance after removal and the sequelae of the procedure (Fig. 8).

**Figure 6.**
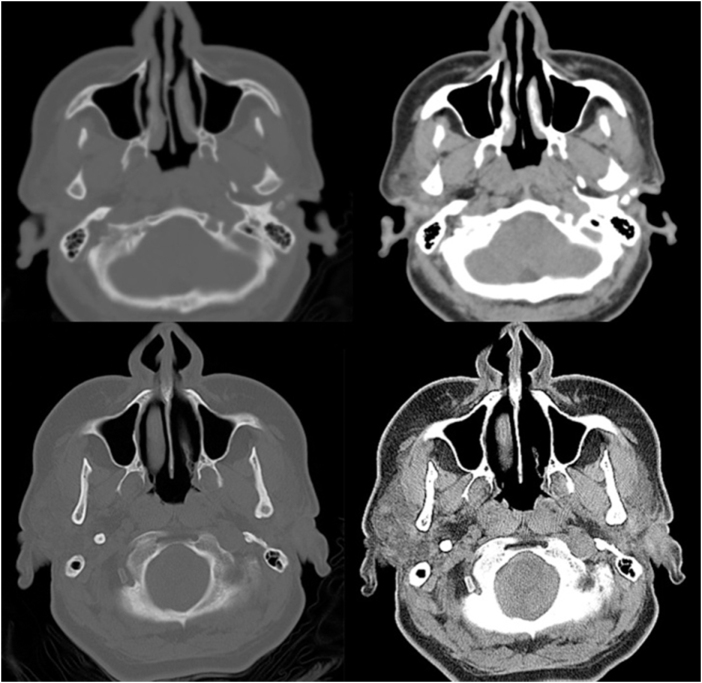
Non-contrast CT demonstrates normal post-operative findings at two years.

**Figure 7.**
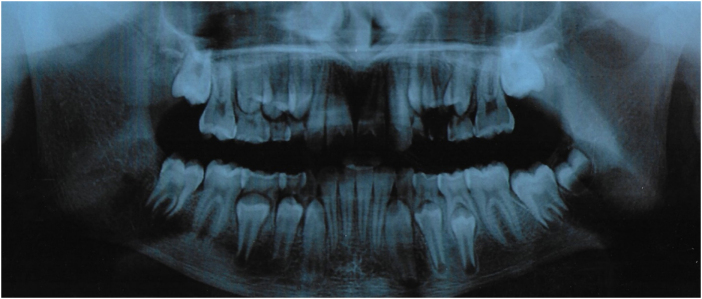
Postoperative panoramic radiograph demonstrating the postoperative appearance.

**Figure 8.**
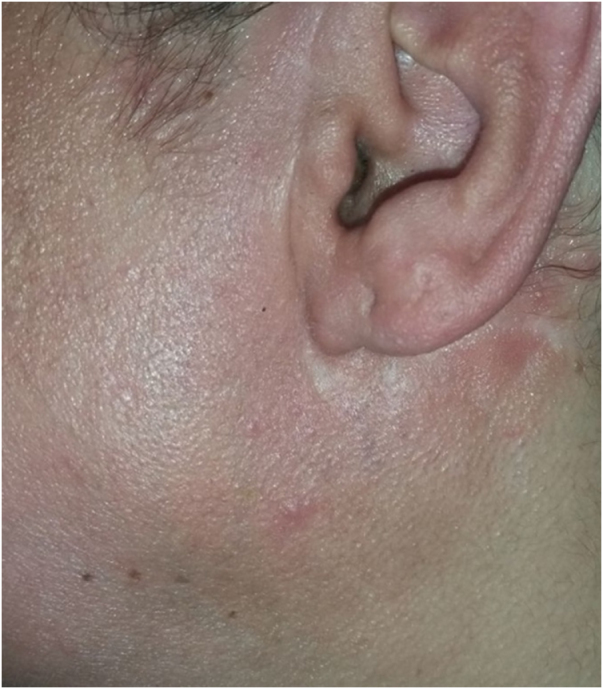
Four-year postoperative status.

### Case 3

A 45-year-old man sustained a penetrating injury to the left mandible and neck from a ballistic fragment, resulting in a mandibular fracture. He underwent fixation of the mandible and maxilla for 35 days, during which a mandibular stent was placed and subsequently removed after 6 months. Five years later, he noticed a firm neck mass with marked swelling and developed hoarseness, most pronounced when turning his head to the left – the side corresponding to the fragment. The patient reported no signs of infection or dysphagia. Concern for a retained neck foreign body prompted imaging, and a non-contrast CT of the neck demonstrated a foreign object near the larynx (Fig. 9). He underwent left neck exploration and removal of the foreign body; a concomitant purulent laryngocele was encountered, which was addressed, and the laryngeal ventricle was sutured. The laryngeal lumen was not entered. Postoperatively, the patient had an uneventful course with no complications, and his hoarseness improved.

**Figure 9.**
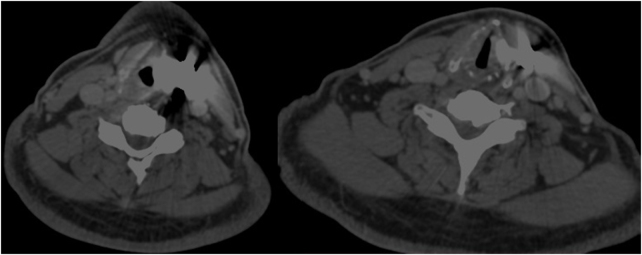
Non-contrast CT demonstrates a left-neck metallic foreign body adjacent to the larynx.

### Case 4

A 34-year-old male farmer presented to the otolaryngology emergency department. The incident occurred while he was working in his field when he suddenly felt something penetrate his neck, accompanied by a small amount of bleeding. He reported no other significant symptoms. Clinical examination revealed a small, few-millimeter wound on the left side of the neck, lateral to the larynx. The patient was in stable condition with no disturbances in vital signs, no active bleeding, and no hoarseness, dyspnea, or dysphagia. No other symptoms were present. Imaging studies, including a plain radiograph and a non-contrast CT scan, showed a metallic foreign body located in the left neck near the larynx (Fig. 10). A neurologists and vascular specialists confirmed the integrity of the vascular and nervous structures. Given the patient’s stable condition, the absence of significant injury, the small size of the foreign body, and the potential risks of surgical intervention due to proximity to the prevertebral fascia, the decision was made to manage the case with observation. The patient was advised to follow up long-term.

**Figure 10.**
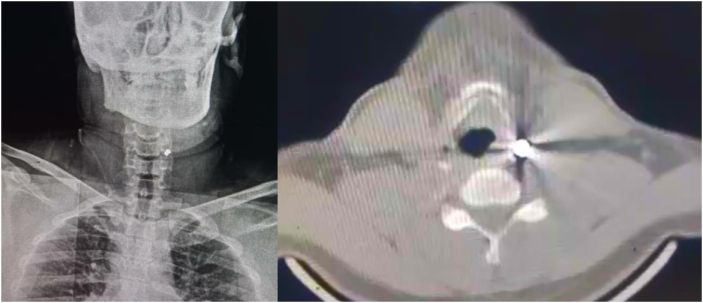
Imaging displaying a metallic foreign body located in the left neck adjacent to the larynx.

## Discussion

Penetrating head and neck injuries with retained foreign bodies present complex clinical scenarios that demand a nuanced, multidisciplinary approach. The four cases presented herein illustrate the spectrum of such injuries, ranging from acute, life-threatening situations to chronic, insidious presentations with delayed complications. These cases underscore the critical importance of tailored diagnostic imaging, careful risk-benefit analysis, and the application of both minimally invasive and open surgical techniques, guided by anatomical and clinical considerations.

The diagnostic cornerstone in all cases was advanced imaging, primarily CT, which provided precise localization of the foreign bodies and delineated their relationship to vital neurovascular structures^[^1,4^]^. This is consistent with the literature, which emphasizes CT as the gold standard for initial evaluation due to its superior ability to detect radiopaque materials and define bony anatomy^[^1,6^]^. In Case 1, CT was instrumental in identifying a fragment within an Onodi cell, a known anatomical variant that increases the risk of optic nerve injury during surgery. The 2-year delay between injury and presentation highlights a key challenge: patients may remain asymptomatic for extended periods, only to present later with complications like chronic inflammation or infection, necessitating a high index of suspicion even in seemingly stable patients^[^1,3^]^. The intraoperative discovery of inflammatory polyps not visible on the initial scan further underscores the dynamic nature of these injuries and the potential for ongoing mucosal changes, reinforcing the need for timely intervention to prevent such sequelae^[^1^]^.

The management strategy must be highly individualized, balancing the risks of retention against the morbidity of removal. As demonstrated in Case 4, conservative management with active observation is a viable and safe option for select patients. This approach is supported by literature that recommends non-operative management for small, inert, asymptomatic foreign bodies located in areas where surgical access would pose a significant risk to surrounding structures, provided there is no evidence of active infection, vascular injury, or neural compromise^[^1,5^]^. The patient’s stability and the confirmed integrity of vascular structures were pivotal in this decision. This conservative paradigm represents an evolution from traditional mandates for exploratory surgery and aligns with modern principles of minimizing unnecessary surgical morbidity^[^5,6^]^.

Conversely, surgical intervention is imperative in cases of symptomatic foreign bodies, infection, proximity to critical structures with the risk of erosion or migration, or functional impairment. The choice of surgical approach is paramount. Case 1 successfully utilized FESS, a minimally invasive technique that offers excellent visualization and access to the sinonasal tract and skull base while minimizing collateral damage^[^6^]^. Its efficacy in retrieving foreign bodies from anatomically complex areas like the sphenoid and ethmoid sinuses, as seen in this case and others in the literature, makes it the preferred first-line surgical modality for centrally located objects^[^6^]^. In contrast, Cases 2 and 3 required open surgical approaches due to the foreign bodies’ locations in the parotid region and deep neck spaces, respectively. The Blair incision in Case 2 allowed for safe dissection and preservation of the facial nerve and Stensen’s duct, structures whose injury could lead to significant long-term morbidity like Frey syndrome, which the patient indeed developed postoperatively^[^1,3^]^. This highlights a critical tenet of head and neck surgery: the surgical corridor must be planned not only to remove the object but also to meticulously identify and protect vital nerves and vessels, often requiring the use of intraoperative navigation or neuromonitoring^[^4^]^.

The variable and often delayed presentation of complications is a recurring theme. Case 2 developed severe neuropathic pain years after the initial injury, likely due to chronic inflammatory changes or fibrosis affecting the trigeminal nerve. Case 3 presented with a neck mass and hoarseness 5 years post-trauma, leading to the discovery of a purulent laryngocele associated with the foreign body. These cases exemplify the “ticking time bomb” nature of retained objects, which can lead to delayed abscess formation, fistula development, nerve compression, or vascular injury^[^1,3^]^. This necessitates not only prompt treatment at presentation but also long-term follow-up for patients with retained fragments to monitor for the emergence of late sequelae.

The integration of a multidisciplinary approach cannot be overstated in the management of complex penetrating neck injuries, particularly those involving impalement or massive tissue destruction, where coordination between otolaryngology, vascular surgery, and neurosurgery is critical to avoid catastrophic outcomes^[^8^]^. The use of advanced imaging and surgical navigation systems has further refined our ability to precisely locate and safely remove foreign bodies in proximity to vital neurovascular structures, thereby reducing operative risks and improving functional preservation^[^9^]^. Additionally, the context of injury significantly influences both the severity and management strategy; some injuries involve high-velocity mechanisms and greater tissue loss, necessitating more aggressive reconstruction and prolonged rehabilitation^[^10^]^. This underscores the importance of tailoring surgical timing and technique to the etiology and extent of trauma, balancing immediate intervention against the potential for delayed reconstruction when appropriate.

These cases reinforce the indispensability of a multidisciplinary team, encompassing otolaryngology, neurosurgery, maxillofacial surgery, radiology, and vascular surgery. Preoperative planning with detailed imaging review, as conducted with the vascular specialist in Case 4, is crucial for risk stratification and formulating a safe surgical plan. For high-risk cases involving the internal carotid artery, as illustrated in the literature, endovascular preparation with standby stenting capabilities can be a lifesaving measure^[^5^]^. This collaborative model ensures that care is comprehensive, leveraging the expertise of each specialty to optimize functional and aesthetic outcomes while mitigating risks.

The management of penetrating head and neck foreign bodies is not a one-size-fits-all endeavor. It requires a sophisticated algorithm based on precise anatomical localization via advanced imaging, a thorough assessment of symptoms and risks, and a tailored approach that ranges from vigilant observation to image-guided minimally invasive or open surgical exploration. The primary goals remain the prevention of life-threatening complications, the preservation of function, and the minimization of long-term morbidity through careful decision-making and technical excellence.

## Conclusion

This case series highlights the critical importance of individualized management in penetrating head and neck injuries with retained foreign bodies. Advanced imaging is indispensable for precise localization and risk assessment. Management must be tailored to each case, ranging from minimally invasive endoscopic retrieval to open surgery or even active observation in selected stable patients. A multidisciplinary approach ensures comprehensive care, optimizing functional and aesthetic outcomes while minimizing complications. Long-term follow-up remains essential to monitor for delayed sequelae, underscoring the need for careful patient selection and meticulous surgical planning.

## Data Availability

The data that support the findings of this study are available from the author or the corresponding author upon reasonable request.
